# A conversation with Dr. David J. Maron, Director of Preventive Cardiology at Stanford Hospital and Clinics

**DOI:** 10.1017/cts.2022.458

**Published:** 2022-12-27

**Authors:** Kathy Siranosian

**Affiliations:** Clinical Research Forum, Washington, DC, USA

## Top 10 Clinical Research Achievement Awards Q & A

This article is part of a series of interviews with recipients of Clinical Research Forum’s Top 10 Clinical Research Achievement Awards. This article is with Dr. David J. Maron, Director of Preventive Cardiology at Stanford Hospital and Clinics. Dr. Maron, along with Dr. Judith Hochman at NYU, designed and carried out the ISCHEMIA (International Study of Comparative Health Effectiveness with Medical and Invasive Approaches) trial over more than a decade. The trial examined the impact of adding invasive procedures to guideline-directed medical therapy for patients with stable coronary artery disease and provides important information to use in decision-making between physician and patient on disease management. This study received Clinical Research Forum’s highest honor in 2021, the Herbert Pardes Clinical Research Excellence Award, as the research study that best shows a high degree of innovation and creativity, advances science, and has an impact upon human disease. *The interview has been edited for length and clarity.*

## How did you become involved in clinical research?

I began medical school with an interest in preventive medicine, and after finishing my residency in internal medicine, I was fortunate to get a postdoctoral fellowship in prevention and epidemiology at Stanford, funded through the Robert Wood Johnson Foundation Clinical Scholars Program. For that 2-year fellowship, I was a member of the investigator team of a National Heart, Lung, and Blood Institute (NHLBI) trial looking at the impact of multiple risk factor intervention on the progression of atherosclerosis in coronary arteries. At first, I was a bit skeptical. As someone who was interested in prevention, I thought I should be working with the pediatric age group or with people who didn't already have heart disease. Well, suffice it to say this turned out to be the perfect assignment for me. I loved being part of a team and that we were asking a critical question: Can the progression of atherosclerosis be slowed by controlling risk factors? In the end, we found that, yes, controlling risk factors could slow disease progression and that, incidentally, there were fewer hospitalizations for cardiac events in this small, single-center trial. Those results then led us to the next question: If controlling risk factors can slow disease progression and reduce cardiac events, how does that compare to stenting? Stenting was a relatively new procedure at the time, and that question led to the COURAGE (Clinical Outcomes Utilizing Revascularization and Aggressive Drug Evaluation) trial [1].

## And it was during these trials that you realized your passion for clinical research?

Yeah, I was hooked. What we found in the COURAGE trial was that adding stents to good medical therapy did not reduce the risk of heart attack and death in people with stable coronary disease. That result led us to the next big question: If you don’t need to insert stents to prevent heart attacks and death, is it necessary to send patients with abnormal stress tests to cardiac catheterization in the first place? That’s what the ISCHEMIA (International Study of Comparative Health Effectiveness with Medical and Invasive Approaches) trial was all about [2].

## As you describe them, these trials flow from one to the other, but each one can take years to complete. What keeps you motivated and engaged?

We started designing the COURAGE trial in the mid-1990s and published the results in 2007 – and a timeframe like that is not unusual at all. These trials can take 10 years or more. That’s why you really need to be passionate about it and determined to find the answer. And you need to be asking important questions. You don't want to spend all that time and end up with a negative result or a result that’s just a little blip, nothing more. I like the idea of asking questions that have an important answer, no matter what the answer will be. With the ISCHEMIA trial, whether the results showed that we can reduce cardiac events with an invasive strategy or that we didn't reduce events, there was going to be an important clinical impact.

## What are the main challenges you had to overcome during the ISCHEMIA trial?

There are decisions and challenges that occur in the pre-award phase of any trial, and these generally revolve around settling on design and getting funding. Then you move on to the post-award phase, where there are different kinds of challenges, such as staying within budget and just meeting the milestones that you or your sponsors set. During the ISCHEMIA trial, we had to overcome three specific challenges [3]. The first centered around recruitment. We ended up reducing the sample size from 8000 to 5000, extending recruitment by 6 months, and extending follow-up by 6 months. We were able to do that and still retain sufficient power to answer the question. The second challenge involved eligibility criteria, and we had to deviate from the original design and accept non-imaging exercise tolerance testing as a qualifying test. The third challenge was a big one and it involved changing the trial’s primary endpoint. We changed from a five-component primary endpoint (cardiovascular death, myocardial infarction (MI), or hospitalization for unstable angina, heart failure, or resuscitative cardiac arrest) in our application to a two-component endpoint (cardiovascular death or MI) when we launched the trial. We built into the protocol that if we didn't have enough events that we could revert to the original five-component endpoint after review by an independent body. Ultimately, we didn't have enough events so we reverted to the original five-component primary endpoint after independent review. We had the foresight to pre-specify a process if necessary to change the primary endpoint. We welcomed a lot of debate about each one of these changes. In the end, NHLBI concurred with our recommendations and we were able to preserve the integrity of the trial.

## What made it possible to overcome these challenges?

When running a clinical trial, there are certain realities that you need to face – realties about time, money, and what’s happening on the ground, in terms of the numbers of patients you are able to enroll, and so on. To deal with these realities and preserve the integrity of the trial, you need to anticipate what kind of problems there might be and have a good process for deliberating with the steering committee. You do not want to sacrifice the ability to answer the question, but you may have to make some sacrifices here and there to be able to complete the trial. With the ISCHEMIA trial, we were fortunate to have a multidisciplinary team that could consider the different perspectives and have an effective deliberative process. It came down to having good communication, good cooperation, and a partnership with NHLBI that was incredibly supportive. We were able to compromise and keep our eyes on the long-term goal, and that resulted in a successful trial. We did not find evidence that the initial invasive strategy reduced the risk of ischemic cardiovascular events or death from any cause. The trial findings were sensitive to the definition of myocardial infarction used.

## What advice do you have for people beginning their careers in clinical research?

The ability to compromise, to get along with other people, to keep in mind the long-term goal, to be passionate about the question you're asking, to be flexible – these are all important qualities to be able to get to the finish line. For clinical research, teamwork is critical. One of the first things I did in beginning the process of planning the ISCHEMIA trial was to reach out to thought leaders in interventional cardiology and ask them to become part of the design team. It’s just so critical to involve important stakeholders in the process. We had a committee for optimal medical therapy, and we added a committee for optimal revascularization therapy, so that we had the expert input from interventionalists and surgeons, so all constituencies were considered in designing the trial and selecting sites. Those really basic lessons you learn as a child, lessons about listening to other people’s perspectives when making decisions, turn out to be important, not just in clinical research, but throughout life.

## Outside of clinical research, what other activities do you enjoy? How do these activities impact your work?

One of my favorite things to do is to play sports, especially racquet sports. I play tennis with friends and colleagues, and recently a friend from the sixth grade introduced me to paddle tennis. Every now and then I’ve been known to sing. Being physically active, having fun, and having a creative release helps keep me balanced and ready for the next challenge.
